# ECM-related gene expression profile in vascular smooth muscle cells from human saphenous vein and internal thoracic artery

**DOI:** 10.1186/1749-8090-8-155

**Published:** 2013-06-18

**Authors:** Tian-xiang Zhu, Bin Lan, Ling-ying Meng, Yan-long Yang, Rui-xiong Li, En-min Li, Shao-yi Zheng, Li-yan Xu

**Affiliations:** 1 Department of Cardiovascular Surgery, Cardiovascular Institute, Affiliated Shantou Hospital of Sun Yat-sen University, Shantou, Guangdong Province 515031, China; 2 Cardiovascular Institute, Affiliated Shantou Hospital of Sun Yat-sen University, Shantou, Guangdong Province 515031, China; 3 Department of Biochemistry and Molecular Biology, Medical College of Shantou University, Shantou, Guangdong Province 515041, China; 4 Guangdong Cardiovascular Institute, Guangdong General Hospital, Guangdong Academy of Medical Sciences, Guangzhou, Guangdong Province 510080, China; 5 Department of Pathology, The Key Immunopathology Laboratory of Guangdong Province, Medical College of Shantou University, Shantou, Guangdong Province 515041, China

**Keywords:** Vascular Smooth Muscle Cells, Restenosis, Gene Expression Profile, Extracellular Matrix, Migration

## Abstract

**Abstract:**

Currently, Saphenous vein (SV) and internal thoracic artery (ITA) are still the most common graft materials in Coronary Artery Bypass Grafting (CABG) whereas SV graft have a lower long-term patency than ITA. Vascular smooth muscle cells (VSMCs) phenotype conversion, proliferation and migration may play a key role in mechanism of vein graft restenosis. To explore differential gene expression profile in VSMCs from SV and ITA will help to further elucidate the mechanism of VSMCs in vein graft restenosis after CABG and to provide new thread of gene therapy.

**Methods:**

VSMCs from paired SV and ITA were cultured for experiments of Affymetrix microarrays and verification using FQ RT-PCR, while the database for annotation, visualization and integrated discovery bioinformatics resources (DAVID 2.0) was utilized for bioinformatics analysis of differential gene expression profile between SV VSMCs and ITA VSMCs. RNA of tunica media from SV and ITA segments were extracted for FQ RT-PCR to display differential expression of PLAT

**Results:**

54,613 probe sets were examined by gene microarray experiments. In SV VSMCs, 1,075 genes were up-regulated and 406 of them were higher than two-fold; 1,399 genes were down-regulated and 424 of them were lower than two-fold as compare with ITA VSMCs.14 ECM-related genes differentially expressed were verificated and listed as following: COL4A4, COL11A1, FN1, TNC, THBS, FBLN, MMP3, MMP9, TIMP3, WNT5A, SGCD were higher whereas COL14A1, ELN, PLAT lower in SV VSMCs than ITA VSMCs. In addition, PLAT was lower in tunica media from SV segments than ITA.

**Conclusion:**

VSMCs from SV and ITA have distinct phenotypes characteristics. Both promoting and inhibiting migration ECM-related genes were higher in VSMCs from SV as compared with ITA, suggesting that VSMCs from SV have more potential migrating capability whereas less PLAT both in SV VSMCs and vascular tissue,implying that SV may prone to be restenosis after CABG.

## Background

Coronary artery bypass grafting (CABG) is one of most effective treatment of coronary heart disease, especially applied in severe patients with multivessel disease and multiple risk factors. Saphenous vein (SV) and internal thoracic artery (ITA) are routinely used grafts in CABG. However, SV grafts exhibit lower patentcy and higher patient mortality as compare with ITA grafts; up to 50% of the SV grafts occlude within 10 years after implantation but rarely of ITA grafts [1]. The difference is probably related to the vascular properties, leading to accelerated atherosclerosis of SV grafts after CABG, whereas resistance of ITA grafts [2].

Restenosis of SV grafts is featured by early thrombosis, intimal thickening in metaphase, and final accelerated atherosclerosis [3]. Vascular smooth muscle cells (VSMCs) phenotype conversion, proliferation and migration play a significant role in the complex pathological process and influence the long-term patency of venous grafts [4]. VSMCs consist of heterogeneous subtypes among various vascular beds and at different vascular developmental stages. VSMCs from veins and arteries have different embryonic origins and exhibit different intrinsic characteristic [5]. Hence, VSMCs from SV and ITA may have distinct intrinsic properties as well, thereby determining patency rates of grafted vessels.

The process VSMCs migration from tunica media to the intima accompanied with extracellular matrix (ECM) remodeling is a dynamic balance of matrix synthesis and degradation [6]. ECM play an important role in the process of VSMCs migration and restenosis. ECM is degraded to form tunnel to facilitate VSMCs migration from tunica media to intima. In addition, ECM components interaction and associated signal transduction participated in restenosis process such as VSMCs phenotype conversion, proliferation and migration. Once ECM secretion and degradation lose the balance, VSMCs proliferaion and migration may be promoted subsequently result in restenosis.

Tissue-type plasminogen activator (PLAT/t-PA) is a significant serine protease associated with ECM degradation and mediates the conversion of plasminogen to plasmin [7]. As the main ingredient of fibrinolytic system, PLAT plays an important role in prevention and treatment of restenosis so that has been widely used in clinical [8]. The endothelium is indeed a rich source of PLAT; loss of the endothelial layer renders fibrinolysis dependent on PLAT released from VSMCs [9]. Deficiency of PLAT may lead to grafts thrombosis and restenosis after CABG.

VSMCs from SV and ITA possess different intrinsic properties and exhibit distinct response to stimuli. VSMCs from SV are more differentiated and show higher contractility whereas prone to proliferation and migration compared to cells from ITA. [3,9,10] The specific mechanisms are still unclear so that comparing differential gene expression profile of VSMCs from SV and ITA will help to further understanding the molecular mechanisms of grafts restenosis after CABG and enlighten new ideas of treatment.

## Methods

Vessels gathering and VSMCs culture This study was approved by Clinical Research Ethics Committee of Affiliated Shantou Hospital of Sun Yat-sen University (2009005). SV and ITA tissue were obtained from 21 patients undergoing coronary artery bypass grafting in Guangdong General Hospital and immediately preserved in -80°C refrigerators. SV and ITA VSMCs were isolated by explant-culture method from fresh specimens. The identity of each VSMCs isolate was confirmed by immunofluorescent staining for SMα-actin (DAKO Corporation). VSMCs of passage 2 ~ 8 endured 48 h serum deprivation were prepared for subsequent experiments.

Cell proliferation assays were taken using MTT kit (keyGEN). VSMCs were divided into 3 groups with differental factors: serum-free DMEM/F12, DMEM/F12 containing 10% FBS or DMEM/F12 containing 10 ng/ml PDGF-BB, and were observed immediately and cultured after 48 h, 96 h, 144 h. Three separate experiments each with 3 replicate wells for each condition were performed for the assays.

### RNA isolation

Total RNA of confluent VSMCs were isolated using TRIZOL reagent (Invitrogen) and the quality was detected by UV Bio-Photometer (Eppendorf). Only sample with a 260/280 nm ratio between 1.9 and 2.1 as well as a 28S/18S ratio between 1.5 and 2.0 were included for further experiments.

-70°C preserved vessels specimen were dislodged standing at room temperature. After adventitia removed and intima scrapped, the remaining tunica media of vessels were rinsing and extracted by grinding in liquid nitrogen. Total RNA of the tissue was isolated and assessed as the same as VSMCs.

### Microarray gene expression profiling and bioinformatics analysis

VSMCs cultured from 3 paired vessels originated from the same patients were selected for the gene microarray experiments. Total RNA was isolated as described and reverse-transcribed using Affymetrix one-cycle cDNA Synthesis Kit, then the cDNA was transcribed to biotin-labeled cRNA using GeneChip IVT Labeling Kit. Biotin-labeled cRNA was fragmented for hybridization to GeneChip Human Genome U133 Plus 2.0 arrays. After 16 h of hybridization, arrays were washed and stained using Genechip fluidics station 450 then scan using gene array scanner 3000. All the process were strictly according to Affymetrix GeneChip Operations Manual.

The raw data was gathered by Affymetrix GCOS 1.4 software (Affymetrix, Santa Clara, CA) with MAS 5.0 algorithm standardization. Fold changes of gene expression difference >2.0 were list for subsequent bioinformatics analysis using DAVID 2.0 (the database for annotation, visualization and integrated discovery bioinformatics resources), including the GO (gene ontology), PA (pathways, including KEGG, BIOCARTA database) analysis. The index of the DAVID and literature *Huang da W* described on Nature Protocols were consulted for analytical methods, and relative recommending values were deployed for the main parameters settings [11].

### Fluorescent quantitation real time polymerase chain reaction

After bioinformatics analysis, 14 ECM-related genes differential expression were verified by fluorescent quantitation real time polymerase chain reaction (FQ RT-PCR). cDNA was synthesized using Reverse Transcription System Kit (Promega) and identified by PCR and agarose gel electrophoresis. Only cDNA exhibiting amplification strap consistent with target gene as well as non primer dimmer was selected for subsequent amplication of 14 ECM-related genes mRNA. The forward and reverse primer (Table 1) synthesized by TAKARA were applied for FQ RT-PCR. The same condition was used for all candidate genes as following: 1 μl of templete cDNA, 5 μl l 2 × PCR Master Mix, 0.2 μl primer F (10 μmol/L), 0.2 μl primer P (10 μmol/L), 3.6 μl RNase-free water by using the following cycling parameters: 95° for 15 seconds for 1 cycle, 95° for 5 seconds, 60° for 15 seconds, 72° for 20 seconds, for a total of 40 cycles. 3 parallel holes were set up for each gene. The data was standardized using β-actin as reference gene for further analysis. 12 paired VSMCs from SV and ITA were taken for the consolidation experiments. 21 SV and 13 ITA segments, including 12 paired samples, were applied for detetion of PLAT.

**Table 1:** PCR primer of 14 ECM-related gene

***Primer name***	***Sequence (5’ → 3’)***	***Length of product***
COL4A4/F	ACCCTGCCAGTCACTTTGGTC	103bp
COL4A4/R	ATACCAGGCAAGCCCTGCTC	
COL11A1/F	AGCTGCAGGCCAAGATGGA	116bp
COL11A1/R	CAATCAGGCCAATTAAACCAGGA	
FN-1/F	GAGCTGCACCTGTCTTGGGAAC	133bp
FN-1/R	GGAGCAAATGGCACCGAGATA	
TNC/F	ACTCGCTACAAGCTGAAGGTGGA	122bp
TNC/R	GCACAGTTGGTGATGGATGAA	
THBS/F	TGCTCCAATGCCACAGTTCC	132bp
THBS/R	CTGCTGAATTCCATTGCCACA	
FBLN/F	CAACCTGCAGCAGCAGACGTGCTA	125bp
FBLN/R	AGCCAGGGTTCTCAGCAGGA	
MMP3/F	GGGTGAGGACACCAGCATGA	178bp
MMP3/R	CAGAGTGTCGGAGTCCAGCTTC	
MMP9/F	ACGCACGACGTCTTCCAGTA	94bp
MMP9/R	CCACCTGGTTCAACTCACTCC	
TIMP-3/F	TGGCCAAGCTGGAGGTCAAC	75bp
TIMP-3/R	CCCGTGTACATCTTGCCATCATAG	
WNT5A/F	TTCGCCCAGGTTGTAATTGAAG	168bp
WNT5A/R	CTGCATGTGGTCCTGATACAAGTG	
SGCD/F	CTTGGCCATGACCATCTGGA	132bp
SGCD/R	GGCAGACTTGAAGTACAGGGCATTA	
COL14A1/F	AAGCCCAGAGTCAAAGTTGTGGA	123bp
COL14A1/R	CCATGAACCATCGACCAGGA	
ELN/F	AGCAAGACCTGGCTTCGGATT	122bp
ELN/R	CCAACGTTGATGAGGTCGTGA	
PLAT/F	TCGAGACTCAAAGCCCTGGTG	119bp
PLAT/R	AGGCTGACCCATTCCCAAAGTAG	
β-actin/F	CAACTGGGACGACATGGAGAAA	178bp
β-actin/R	GATAGCAACGTACATGGCTGGG	

### Statistics

For disparate experiment, VSMCs from same or different patients were used. Accordingly, statistical evaluation was performed by paired or independent nonparameter test: Wilcoxon Signed Ranks Test or Mann–Whitney Test as appropriate. A P-value < 0.05 was considered statistically significant.

## Results

### Cell identification and cell proliferation assay

**Figure 1:**
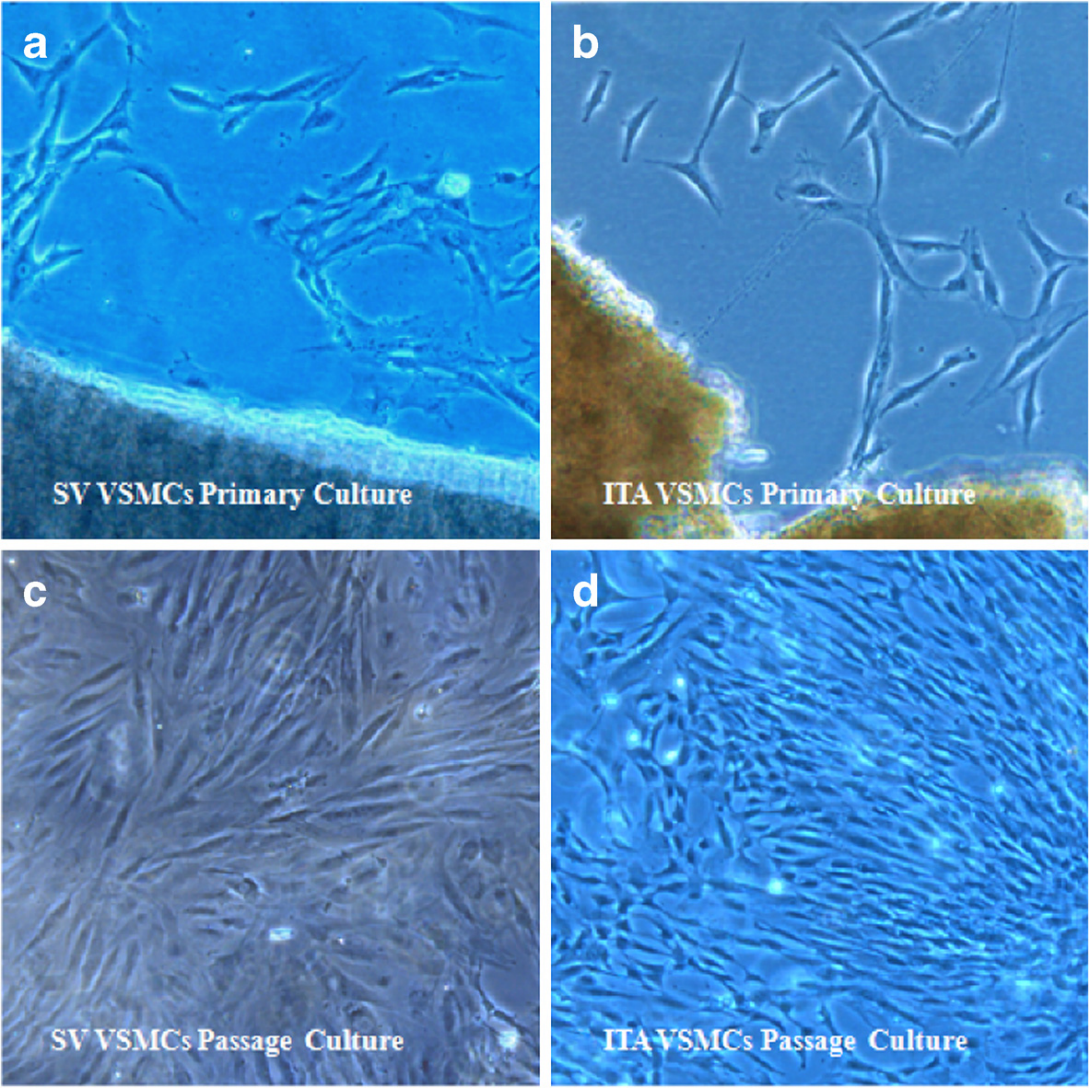
**Morphous of SV VSMCs & ITA VSMCs. a)**. SV VSMCs emigrated from the tissue explant (10 × 10). **b)**. ITA VSMCs emigrated from the tissue explant (10 × 10). **c)**. SV VSMCs grew form a typical “hill and valley” pattern (10 × 5). **d)**. ITA VSMCs grew form a typical “hill and valley” pattern (10 × 5).

**Figure 2:**
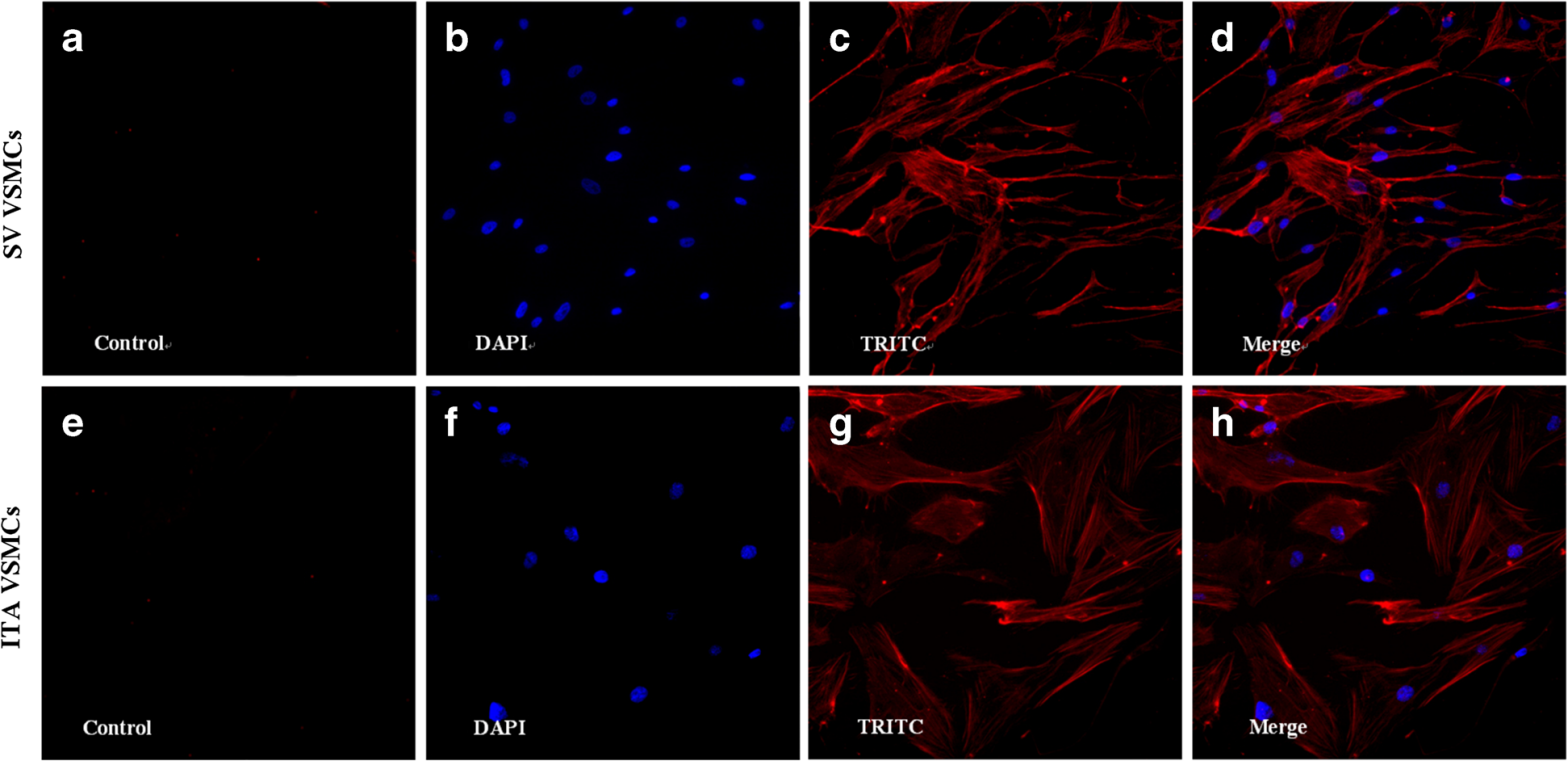
**Identify SV and ITA VSMCs by immunofluorescence staining (10 × 20).** SV VSMCs: **a)**. Control **b)**. DAPI display nucleolusc. **c)**. TRITC display SMα-actin. **d)**. Merge panel. ITA VSMCs: **e)**. Control. **f)**. DAPI display nucleolusc. **g)**. TRITC display SMα-actin. **h)**. Merge panel.

VSMCs were cultured (Figure 1) and identified by immunofluorescence using DAPI labeled nuclei and TRITC marked SMα-actin in the cytoplasm. The cells > 95% purity were selected for subsequent experiments (Figure 2).

**Figure 3:**
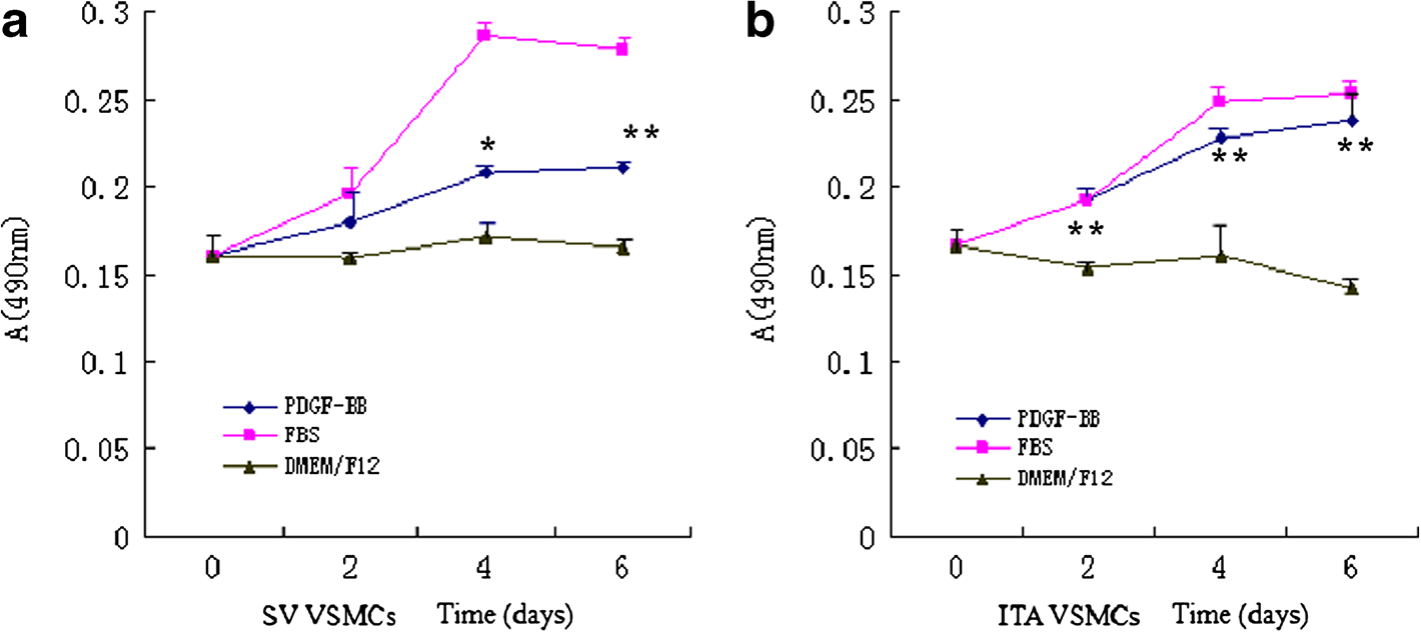
**Proliferation assay of VSMCs from SV & ITA using MTT Kit. a)**. growth curve of SV VSMCs: **b)**. growth curve of ITA VSMCs. P-value: obtained by paired *t*-test between group PDGF-BB and group DMEM/F12 using SPSS 13.0 (*P < 0.05; ** P < 0.01).

**Figure 4:**
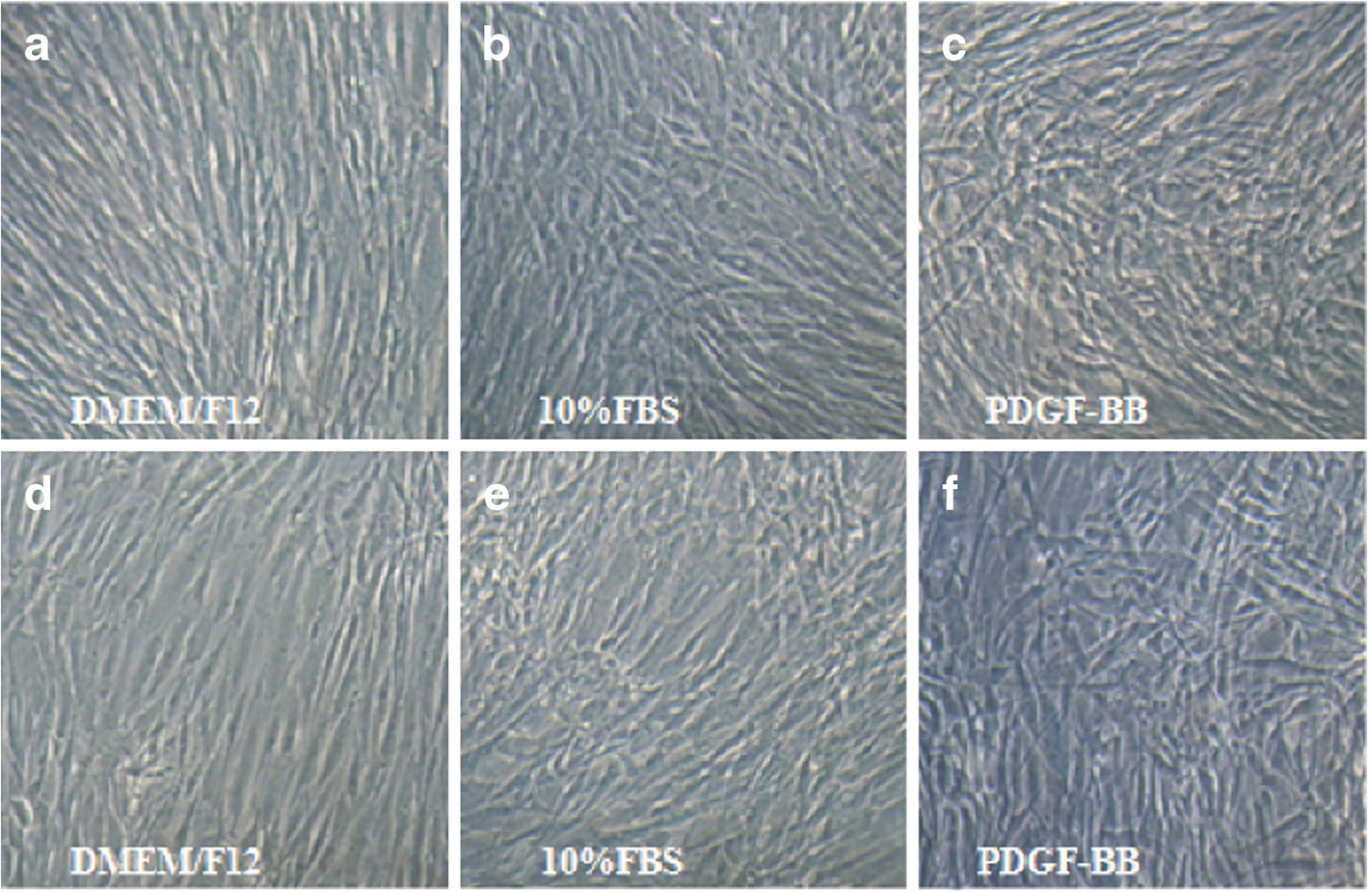
**Morphology diversity under distint treated factors (Magnification 10 × 10).** SV VSMC: **a)** DMEM/F12 **b)** 10%FBS **c)** 10 ng/ml PDGF-BB. ITA VSMC: **d)** DMEM/F12 **e)** 10%FBS **f)** 10 ng/ml PDGF-BB. **a)** and **d)**: Cells grew with long and thin shape, refractive index of the cell edge increased. Cells line more compactly, and gradually formed a multil-overlapping, bundles, polarity, similar to the organic assembled VSMCs of the blood vessel wall. **b)** and **e)**: Cells grew with typical "hill and valley" pattern, from multil-overlapping and non-cell regions. **c)** and **f)**: Polarity of the cells disappeared, and proliferation were enhanced,form many ultra-overlapping regions.

VSMCs cultured in medium with different factors displayed distinct cell growth curve (Figure 3). Both VSMCs from SV and ITA exhibited intense responsibility to FBS and PDGF-BB with dramatic proliferation reacting to stimuli.(Figure 4) In SV VSMCs, the data detected after 96 h and 144 h between PDGF-BB and DMEM/F12 was statistically significant. (P < 0.05 or P < 0.01). In ITA VSMCs, the data detected after 48 h, 96 h and 144 h between PDGF-BB and DMEM/F12 was statistically significant. (P < 0.01).

### Microarray gene expression profiling and bioinformatics analysis

**Figure 5:**
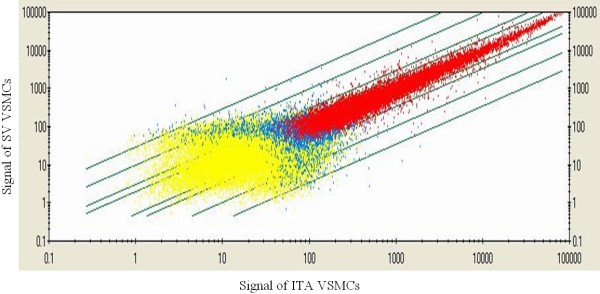
**Scatter graph of microarray analysis of gene expression.** Fold change Line from diagonal to two side represent 2,3,10,30 fold respectively. Yellow color: Low expression in VSMCs both ITA & SV. Red color: High expression in VSMCs both ITA & SV. Blue color: Differential expression in VSMCs from ITA & SV.

**Figure 6:**
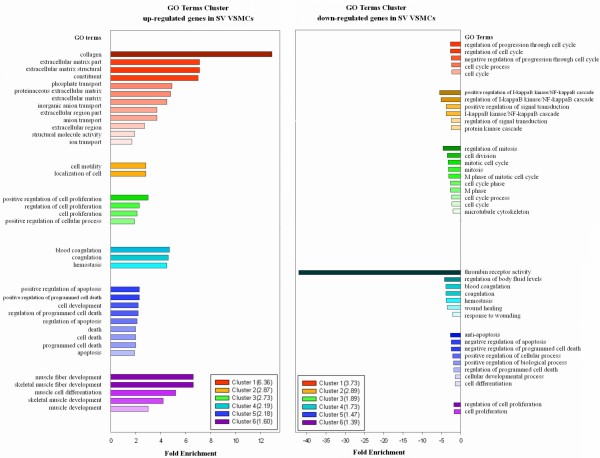
**GO terms cluster of up-regulated and down-regulated genes in SV VSMCs.***Fold Enrichment (FE):* To measure the magnitude of enrichment. For example, 10% of analysis genes are kinases versus 1% of genes in human genome (this is population background) are kinases. Thus, the fold enrichment is tenfold. Fold enrichment 1.5 and above are considered as statistical significance. Negative value means that the GO term was down-regulated in SV VSMCs. *GO Terms:* A detailed GO item in the annotation source of DAVID system. Each set of color bars represents a annotation term group(cluster). Values in brackets was *Enrichment Score (ES)* of each cluster : To rank overall importance (enrichment) of annotation term groups. It is the geometric mean of all the enrichment P-values (EASE scores) of each annotation term in the group. To emphasize that the geometric mean is a relative score instead of an absolute P-value, minus log transformation is applied on the average P-values. ES > 1.3-fold (equivalent to non-log scale 0.05) was considered as statistical significance, mean that the gene cluster was significantly enriched in analysis list. *P-value (or called EASE score, for individual term members):* To examine the significance of gene–term enrichment with a modified Fisher’s exact test (EASE score). P <0.05 was considered as statistical significance.

54,613 probe sets were examined by gene microarray experiments and the differential gene expression profile of VSMCs from SV and ITA was processed for further bioinformatics analysis. Scatter Graph of microarray experimental data shown that the majority genes expression in SV VSMCs consistent with ITA VSMCs and differentially expressed genes accounted for a small part. (Figure 5) In SV VSMCs as compared with ITA, 1,075 genes were up-regulated including 406 gene higher than two-fold; 1,399 genes were down-regulated including 424 lower than two-fold. Differential gene expression profile was analyzed using Gene Functional Classification and exhibited that 27 gene clusters were up-regulated while 17 gene clusters were down-regulated in SV VSMCs and 6 representative gene clusters of both category were selected and shown (Figure 6). Differentially expressed genes terms covered VSMCs phenotypic markers, proliferation, extracellular matrix (ECM), apotosis/anti-apoptosis, cell cycle, coagulation, IGF binding protein and other GO terms and various signal transduction pathways(PA), such as ECM-receptor interaction, p53, TGF-beta, Jak-STAT(janus kinase/signal transducer and activator of transcriptase), cell cycle and fibrinolysis pathways.

### ECM-related genes were differentially expressed in VSMCs from SV and ITA

**Figure 7:**
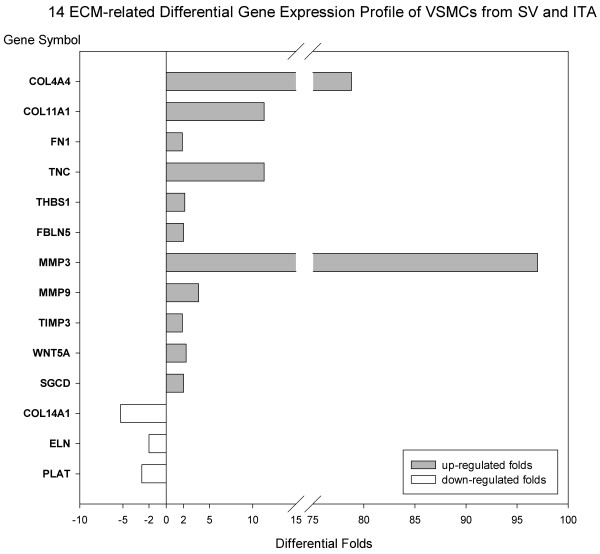
**14 ECM- related gene expression profile of VSMCs from SV and ITA.** Differential Folds: The median ratio of gene expression in VSMCs from SV and ITA. Negative value mean that the gene was down-regulated in VSMCs from SV as comparing with ITA.

**Figure 8:**
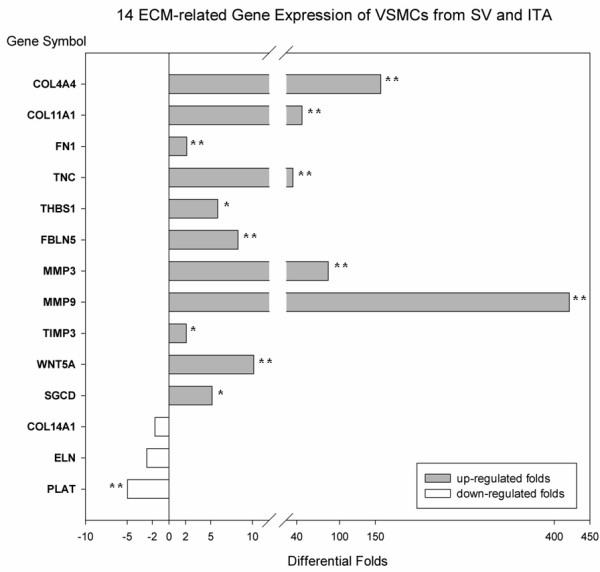
**Differential expression of 14 ECM-related Genes of VSMC from SV and ITA (n = 12) (FQ RT-PCR).** Differential Folds: The median ratio of gene expression in VSMCs from SV and ITA. Negative value mean that the gene was down-regulated in VSMCs from SV as comparing with ITA. (* P < 0.05; ** P < 0.01).

14 differential expressed ECM-related genes profile were shown (Figure 7) and consolidation of microarray data carried out by FQ RT-PCR were well consistent with microarray analysis. Among 14 ECM genes, 11 genes were up-regulated in the SV VSMCs: COL4A4, COL11A1, FN1, TNC, THBS, FBLN, MMP3, MMP9, TIMP3, WNT5A and SGCD; whereas 3 genes were down-regulated: COL14A1, ELN and PLAT (Figure 8).

### PLAT was down-regulated in SV tissue as compared with ITA

**Figure 9:**
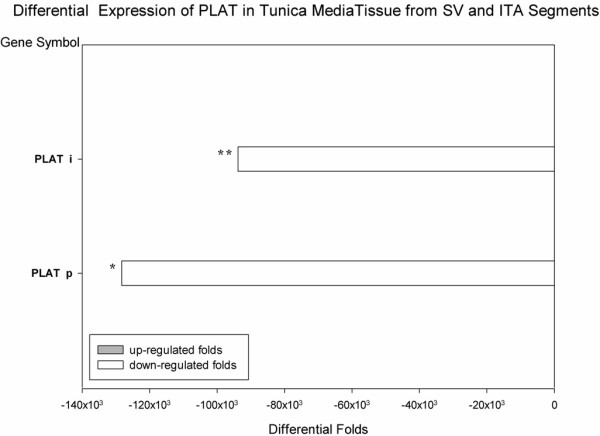
**PLAT was differentially expressed in VSMCs and vessel segment of SV and ITA (FQ RT-PCR).** Differential Folds: The median ratio of gene expression in VSMCs from SV and ITA. Negative value mean that the gene was down-regulated in VSMCs from SV as comparing with ITA. PLAT i: from 21 SV and 13 ITA segments; PLAT p: from 12 paired SV and ITA segments. (** P < 0.001; * P = 0.028).

21 cases of SV and 13 cases of ITA tissue including 12 paired SV and ITA from same patients were selected for RNA isolation for FQ RT-PCR. The data of unpaired or paired tissue were analyzed respectively and chorusly revealed that PLAT was dramatically down-regulated in SV tissue, while compared with ITA. (P < 0.001 and P = 0.028) (Figure 9).

## Discussion

This study demonstrates that SV VSMCs and ITA VSMCs have different patterns of gene expression. Global gene expression profile of VSMCs from SV and ITA reveal different gene expression patterns between venous and arterious grafting conduits for CABG. VSMCs from SV and ITA in vitro exhibited distinct molecular subtypes. As reported, compared with the ITA VSMCs, SV VSMCs were more differentiated, as well as stronger potentiality of proliferation and migration [10].

Differentially expressed ECM-related genes in VSMCs from SV and ITA may play a significant role in the process of VSMCs proliferation, migration and restenosis after CABG. As the major extracellular matrix component of vessel wall and the substrate of MMPs and other protease, collagen regulated VSMCs proliferation and migration through cell-matrix interaction as binding with cell surface receptors and other ECM components, such as tyrosine kinase receptors, fibronectin and integrin [12]. VSMCs from saphenous vein and coronary artery had very different expression of collagen both in basic or pathological state, suggesting that collagen may not only involved in differentiation but also in proliferation and migration of VSMCs [13]. In injured vascular and atherosclerotic lesions, VSMCs synthesized more collagen and adjusted the microenvironment to faciliate VSMCs migration [14]. Our study showed that a variety of collagen were differntially expressed in VSMCs from SV and ITA, correlated with different characters and distinct responds to stimuli between them. Various collagen assign tenacity to tissue toughness and different polymerized types have respective function. COL4, as major component of basal membrane, is one of the main barriers of cell migration. Once they were degradated by collagenase may lead to decollement of basal membrane and accelerated migration of VSMCs [8,15]. COL11 indirectly produced a marked effect in the migration of VSMCs through COL1/2 by changing the hardness of the matrix. COL14, with aggregating collagen fibers as main function, is widespread in connective tissue especially in the higher mechanical tension parts of cambium but less in mature organizations [16]. In our study, COL4A4, COL11A1 expression were up-regulated while COL14A1 down-regulated in SV VSMCs, indicated less migration of SV VSMCs under physiological conditions may be related to tenacity of matrix in basal membrane. Additionally, down-regulation of COL14A1 in SV VSMCs indicated that SV was well differentiated tissue.

Elastin (ELN) around VSMCs in the vessel wall endued organizations flexibility and stabilized the vessel wall by inhibiting the migration of VSMCs, in other words, decrease of ELN may promote the migration of VSMCs [17]. As previous discussion, collagen content could inhibit VSMCs migration. Accordingly, the ratio between elastin and collagen labeled feature of vascular wall and it could be regulated by blood flow, concretely less ratio between elastin and collagen always accompany with slower flow [18]. The migration of VSMCs maintain a balance under precise regulation of both elastin and collagen. In SV under physiological conditions, less ratio between elastin and collagen in the structure accompanied with slower blood flow. Our experiments confirmed this view by less ELN, more COL4 and COL11 in SV. Moreover, VSMCs in SV may be promoted by down-regulation of ELN while inhibited by up-regulation of collagen, hint that they proned to remodeling under definite condition because of the balance in high level.

FN1, TNC, THBS and FBLN are four ECM proteins that play a role through integrin receptors in regulation of cell survival, proliferation and migration through downstream PKC, PI3K, RHO and other pathways [17]. Suppression of FN polymerization or blockade its connection with VSMCs could inhibit VSMCs migration and proliferation [19]. TNC could faciliated reorganization of cytoskeleton system accordingly promoted intima thickening and VSMCs migration from tunica media after arterial injury [20]. But once it was decomposed by MMPs may leading to inhibition of VSMCs proliferation and apoptosis induction [21]. THBS indirectly participated in the migration of VSMCs as a members of cytokines downstream signaling pathways [22]. In summary, FN1, TNC and THBS binded with integrin receptor and carried out cell migration functions through downstream signal transduction [23]. Conversely, FBLN5 could not only organize ELN network to stable VSMCs in the ELN-rich regions, but also combined with extracellular superoxide dismutase and facilitated it bind with vascular tissue to protect the vessel wall [24,25]. In this study, FN1, TNC, THBS were raised as migration promoter factor while FBLN was also increased as inhibitor in SV VSMCs, all of them hold the balance in high level to maintain stability of VSMCs migration. Consequently SV VSMCs may prone to migrate as responding to stimulus.

VSMCs migration to the intima along with ECM remodeling are results of dynamic balance of matrix synthesis and degradation and associated with matrix metalloproteinases (MMPs) and their inhibitors [6]. Various MMPs have been found in vascular tissue, such as MMP3 (stromelysin-1, matrilysin 1), MMP9 (gelatinase B), and respectived inhibitors TIMPs [26]. MMP9, synthesized by VSMCs and macrophages in impaired area, was upregulated along with MMP3 in vascular restenosis and other pathological processes to promote VSMCs phenotypic conversion. MMP9 can promote VSMCs migration to the intima by degrading basal membrane components including collagen type IV, laminin and elastin [26,27]. Potential growth factors and cytokines may be activated and released after MMP9 having degraded the extracellular matrix, and may combine with the ECM components to further faciliation of VSMCs phenotype conversion [28]. MMP9 expression block though small RNA technology may significantly reduce VSMCs migration and intimal thickening [29]. TIMP3 transfection could decrease about 84% intimal thickening in human SV and 58% in pig SV. TIMP3 could not only inhibite migration though decreasing MMPs, but also lead to apoptosis of VSMCs [29]. MMPs and their inhibitors within subtile balance played antagonistic effect in the process of restenosis. Our experiment displayed that all of MMP3, MMP9 and TIMP3 were increased in SV VSMCs, suggesting that MMPs and TIMP maintained a high level balance in SV under physiological conditions, once breaked by pathological fators may lead to rapid progress of disease.

Secreted glycoprotein WNT was a important signaling molecules of ECM, combined with the receptors (frizzled) to produce a marked effect mainly through the second messenger β-Catenin. In rat carotid artery injury model, β-Catenin was significantly increased 7 days after arterial injury to inhibit VSMCs apoptosis and promote their survival through cyclin D1 protein and p21 the cell cycle [30]. SGCD (sarcoglycan complex D) was one of the components of DGC complex (dystrophin-glycoprotein complex), which mediated connection of cytoskeleton F-actin and extracellular matrix component Laminin to play a role in mechanotransduction mechanisms, also mediated signal transduction [31]. It is not very clear that the detailed effect SGCD and DGC in migration of VSMCs, but it can be supposed they associated with cell migration because of their structure specificity . Upregulated of WNT signaling and SGCD along with increased ECM-receptor interaction as a result of 14 differentially expressed ECM-related genes in SV VSMCs implied that SV VSMCs may be prone to ECM remodeling as compared to ITA VSMCs.

In SV VSMCs as compared with ITA, 3 folds main balance in high level correlated with VSMCs migration are as the following: (1) COL4A4 and COL11A1 were higher where as ELN lower. Up-regulation of collagen could inhibit the migration of VSMCs but the reduction of ELN could promote the migration of VSMCs. (2) FN1, TNC and THBS along with FBLN were higher. The former three adhesion molecules could cooperate to promote cell migration whereas FBLN could inhibite migration and stabilize the vessel wall. (3) Not only MMP3, MMP9 but also TIMP3 were higher. MMP3, MMP9 could promote cell migration, whereas their specific inhibitor TIMP3 was also increased to antagonize them. Various ECM-related genes promoting and inhibiting migration simultaneously changed and maintained balance in higher level in SV VSMCs as compare with ITA, once the balance was broken by etiological factors may lead to rapid pathogenic progress, including restenosis after CABG.

Tissue-type plasminogen activator (PLAT/t-PA), mainly produced in endothelial cells, can activate plasminogen to degrade fibrin consequently be an important part of fibrinolytic system in the blood. However, it was more dependent on VSMCs when endothelial layer injury had occured [9]. PLAT played an important role in coronary heart disease through its effective anticoagulation, and according to statistics restonosis occured in 14.4% vein grafts detected by coronary angiography immediately after off-pump CABG. Construction of PLAT transfection model could effectively prevent early stage restonosis after CABG operation [32]. It was already found that PLAT was lower in human SV than ITA, and PLAT protein was lower in supernatant of SV VSMCs cultures. In our study, PLAT was lower both in SV VSMCs and tunica media tissue, consistent with the findings of *Payeli SK [*[9]. Therefore, SV may be prone to generate thrombosis and neointimal formation, which caused restenosis after CABG, whereas ITA had potential antithrombotic ability thereby maintained revascularization.

## Conclusions

VSMCs from SV and ITA have distinct gene expression profile. Both promoting and inhibiting migration ECM-related genes were higher in VSMCs from SV as compare with ITA suggesting that VSMCs from SV have more potential migrating capability. Less PLAT expression both in SV VSMCs and vascular tissue implied that SV may be prone to generate thrombosis and neointimal formation, which caused restenosis after CABG, whereas ITA had potential antithrombotic ability thereby maintained revascularization.

Accordingly, ITA should be kept being strongly recommended to be grafted to anterior descending coronary artery or dominant coronary vessel in CABG for higher patentcy. Furthermore, appropriate gene therapy, including PLAT transfection, probably reduce SV grafts restenosis and benefit patients more after CABG in the future.

### Consent

Written informed consent was obtained from the patient for publication of this report and any accompanying images.

## Abbreviations

SV: Saphenous vein; ITA: Internal thoracic artery; CABG: Coronary artery bypass grafting; VSMCs: Vascular smooth muscle cells; ECM: Extracellular matrix; PLAT/t-PA: Tissue-type plasminogen activator; DAVID: The database for annotation, visualization and integrated discovery bioinformatics resources; GO: Gene ontology; PA: Pathways; FQ RT-PCR: Fluorescent quantitation real time polymerase chain reaction; ELN: Elastin; MMPs: Matrix metalloproteinases; SGCD: Sarcoglycan complex D; DGC complex: Dystrophin-glycoprotein complex.

## Competing interests

The authors declare that they have no competing interests.

## Authors’ contributions

TZ carried out vessels gathering, VSMCs culture, RNA isolation, microarray gene expression profiling and bioinformatics analysis and statistical analysis, participated in FQ RT-PCR and drafted the manuscript. BL conceived of the study, and participated in its design and coordination and helped to draft the manuscript. LM carried out FQ RT-PCR and participated in preliminary experiment and statistical analysis. YY participated in FQ RT-PCR. RL participated in vessels gathering. EL participated in its design and coordination and helped to draft the manuscript. SZ participated in its design and coordination. LX participated in its design and provided directions of empirical method. All authors read and approved the final manuscript.
